# Endothelial dysfunction predicted cardiovascular events in patients with paroxysmal atrial fibrillation

**DOI:** 10.15537/smj.2022.43.7.20220214

**Published:** 2022-07

**Authors:** Jing Zhang, Qiang Tan, Wang Lina, Zhang Zhaoqian

**Affiliations:** *From the Department of Cardiology, Qinhuangdao First Hospital, Hebei Medical University, Qinhuangdao, China*.

**Keywords:** paroxysmal atrial fibrillation, endothelial function, flow mediated dilation, heart failure, cardiovascular event

## Abstract

**Objectives:**

To examine the relationship between endothelial dysfunction and adverse cardiovascular events in patients with paroxysmal atrial fibrillation (AF).

**Methods:**

In this prospective observational study, flow-mediated dilation (FMD) was measured by ultrasound in 291 patients with paroxysmal AF. Then, the patients were divided into low FMD group (n=97, FMD of <5.9%) or high FMD group (n=194, FMD of ≥5.9%). Patients were followed up for at least 30 months. Primary end point was cardiovascular events (stroke, heart failure hospitalization, cardiovascular death, and non-fatal myocardial infarction) and second endpoint was heart failure hospitalization, or stroke.

**Results:**

Rate of cardiovascular events was higher in low FMD group compared with high FMD group (37.1% versus 18%, *p*<0.001). This higher risk of cardiovascular events in patients with low FMD was primarily due to a higher risk of heart failure hospitalization compared with patients with high FMD (19.6% versus 10.8%, *p*<0.05). There was no significant difference of stroke between both groups. Cox proportional hazards ratio (HR) analysis showed that low FMD (HR: 3.036, 95% CI: [1.546-5.963], CHA2DS2-VASc scores (HR: 1.383, 95% CI: [1.035-1.847]), and left atrial diameter (HR: 1.304, 95% CI: [1.001-1.069]) were independent predictors for composite cardiovascular events.

**Conclusion:**

In patients with paroxysmal AF, endothelial dysfunction was associated with composite cardiovascular events. Flow-mediated dilation was a significant predictor of cardiovascular events in patients with paroxysmal AF.

**A**s a most common cardiac arrhythmia in the world, atrial fibrillation (AF) is associated with high risk of mortality and morbidity including stroke, heart failure, and embolic events.^1^

Endothelium not only regulates vasodilation and vasoconstriction, but also has paracrine ability and anti-inflammatory activity.^2^ Endothelial dysfunction is considered to play a key role in pathogenesis of atherosclerosis, hypertension, and heart failure. More importantly, endothelial dysfunction is a contributor to cardiovascular disease, not just a marker.^3^ Our previous study and other studies demonstrated that permanent and paroxysmal AF impaired endothelial function.^4-6^ However, the association of endothelial dysfunction and cardiac events such as stroke, heart failure, and myocardial infarction in patient with AF has not been fully clarified.

Flow-mediated dilation (FMD) is an accurate non-invasive method to evaluate endothelial function.^7^ Flow-mediated dilation could assess the ability of peripheral arteries dilation in response to reactive hyperemia. Previous studies had testified that measurement of peripheral endothelial function by FMD was correlated with coronary artery endothelial function.^8,9^ The aim of this study is to evaluate the association between peripheral vascular endothelial function, assessed by FMD and its long-term outcomes in patients with paroxysmal AF.

## Methods

In this prospective observational study, we followed the methods of our previous study.^10^ Patients with paroxymal AF who scheduled for regular clinical examination in Qinhuangdao First Hospital, Qinhuangdao, China, between October 2014 and September 2018 were enrolled. Inclusion criteria were the following: 1) patients with a history of initial AF, which is defined as the first electrocardiogram (ECG) or Holter monitoring confirmed occurrence of AF; 2) the history of paroxysmal AF was no longer than 6 months; 3) patients with left ventricular ejection fraction (LVEF) of >50%; and 4) aged 25-80 year. Exclusion criteria were as follows: 1) patients with acute myocardial infarction; 2) patients with persistant or permanent AF; 3) patients with valvular heart disease which needs surgical or interventional indications; and 4) patients with a history of heart failure. A total of 357 patients finally enrolled, 291 patients finished follow-up. This study was approved by the Ethics Committee of Qinhuangdao First Hospital and informed consent of patients was obtained before participation.

The types of AF were defined as the following: paroxysmal AF was defined as terminating within 7 days of onset; persistent AF was defined as being sustained longer than 7 days; and permanent AF was defined as a decision to stop attempting to restore or maintain sinus rhythm.^10^

Similar to the methods used in our previous study, FMD was determined by a VIVID 8 ultrasound system (GE Medical Systems).^11^ After baseline brachial artery diameter was measured, a forearm blood pressure cuff was inflated to 225 mmHg for 5 minutes. Ultrasound images of the brachial artery were recorded to obtain end-diastolic pre-occlusion baseline diameter and to determine the maximum post-occlusion brachial artery diameter. Flow-mediated dilation was determined as % change {[(maximum - baseline diameter)/baseline diameter]×100}.^11^

Patients were followed-up for at least 30 months. Patients of both groups were followed up every 6 months by clinic visits. If patients experienced symptoms suggesting cardiovascular events between scheduled visits, they were instructed to contact the study physicians. At each visit, a 12-lead ECG and a list of ongoing medications was obtained. Echocardiography was tested at baseline and 24 months follow-up to evaluate left atrial diameter (LAD), left ventricular diameter, and LVEF. All procedures and analyses were carried out by an experienced researcher who was blinded to the FMD results.

The primary endpoint was composite adverse cardiovascular events, which included cardiovascular death, nonfatal myocardial infarction, stroke, and heart failure hospitalization. The second endpoint was heart failure hospitalization or stroke. All the events were confirmed by the review of medical records by 2 cardiologists who were blinded to the FMD results.

### Statistical analysis

All statistical analyses were carried out by the Statistical Package for the Social Sciences, version 17.0 (SPSSInc., Chicago, IL, USA). Continuous variables were expressed as mean ± standard deviation (SD) of the mean and compared by unpaired Student t-test. Categorical variables between both groups were compared by Chi-square (χ^2^) statistics or Fisher’s exact test. Receiver-operator characteristic curve analyses were carried out to assess the sensitivity and specificity of FMD measurements for predicting cardiovascular events. We divided the patients into 2 groups according to the cut-off values of FMD. Cut-off values were determined according to the highest Youden index from the receiver-operator characteristic curves for predicting the primary outcomes. Time-to-event end point analyses were carried out by using the Kaplan-Meier method. The log rank test was used to compare the groups. Cox proportional hazards regression analysis was carried out to estimate the hazard ratios (HR) and their 95% confidence intervals (CI) of developing the primary endpoint. Variables with a *p*-value of <0.10 in the univariate analysis were chosen in a multivariate analysis. A probability *p*-value of < 0.05 was considered significant.

## Results

The enrolled patients were 357 cases, 66 cases were lost in follow-up period. The final study population comprised 291 paroxymal AF patients, 197 (67.7%) men and 94 (32.3%) women, mean age of 66.34±12.11 years (range 31-80). Patients were divided into low FMD group (FMD<5.9%, n=97) and high FMD (FMD≥5.9%, n=194). Baseline clinical characteristics are shown in Table 1. The 2 groups were balanced in gender, hypertension, smoking, diabetes mellitus, and medication. There were no significant differences in laboratory characteristics such as cholesterol, triglycerides, low density lipoprotein-cholesterol, high-density lipoprotein cholesterol, glucose, and creatinine. However, mean ages of low FMD group were older than that of high FMD group. Echocardiography parameters showed that low FMD group had bigger LAD and lower ejectin traction values. Patients of low FMD group also had higher CHA2DS2-VASc scores and higher level of homocysteine than patients of high FMD group.

**Table 1:** - Clinical characteristics in the 2 groups.

Characteristics	Low FMD (n=97)	High FMD (n=194)	*P*-value
Age	70.14±10.52	64.14±11.01	0.000
Gender (M/F)	66/31	131/63	0.929
Smoking	27	62	0.472
Diabetes	26	69	0.133
Hypertension	32	71	0.544
CHD	29	57	0.928
CHA2DS2-VASc score	3.19±0.97	2.33±0.90	0.000
LVD (mm)	51.53±7.22	50.54±7.51	0.314
LAD (mm)	44.53±7.81	42.27±7.62	0.019
LVEF (%)	59.42±12.28	62.88±9.66	0.017
TC (mmol/L)	4.16±1.19	4.27±1.06	0.472
TG (mmol/L)	1.43±0.90	1.68±1.64	0.117
LDL-C (mmol/L)	2.39±0.91	2.52±0.83	0.220
HDL-C (mmol/L)	1.06±0.28	1.07±0.26	0.869
Glucose (mmol/L)	6.17±1.85	5.85±1.79	0.193
Creatinine (µmol/L)	85.03±43.21	78.52±37.75	0.221
Homocysteine (mmol/L)	19.37±9.95	16.54±7.79	0.021
FMD (%)	4.93±0.84	8.03±1.71	0.000
Wafarin user	33	56	0.368
NOAC user	11	22	0.584
Beita blocker user	72	165	0.359
Diuretics user	11	22	0.584
ACEI/ARB user	31	62	0.389

Values are presented as numbers (n) and mean ± standard deviatin (SD). FMD: flow-mediated dilatation, M: male, F: female, CHD: coronary artery disease, CHA2DS2-VASc: congestive heart failure, hypertension, age ≥75 (doubled), diabetes, stroke (doubled), vascular disease, age 65-74, and gender category (female), LVD: left ventricular diameter, LAD: left atrial diameter, LVEF: left ventricular ejection fraction, TC: totol cholesterol, TG: triglyceride, LDL-C: low density lipoprotein-cholesterol, HDL-C: high-density lipoprotein cholesterol, NOAC: non-vitamin K antagonist oral anticoagulant, ACEI: angiotension converting enzyme inhibitors, ARB: angiotensin receptor blocker

In a mean follow-up of 33±3.7months, 81 patients had cardiovascular events (primary composite endpoints) (Table 2). Rate of cardiovascular events was higher in patients with low FMD compared with patients with high FMD (37.1% versus 18.0%; *p*<0.001). This higher risk of cardiovascular events in patients with low FMD was primarily due to a higher risk of heart failure hospitalization compared with patients with high FMD (19.6% versus 10.8%; *p*<0.05). The rate of cardiovasular death, nonfatal myocardial infarction, and stroke had no significant difference in both groups.

**Table 2:** - Clinical outcomes in the 2 groups.

Variables	Low FMD (n=97)	High FMD (n=194)	*P*-value
Cardiovascular events	36 (37.1)	35 (18.0)	0.000
Heart failure	19 (19.6)	21 (10.8)	0.041
Nonfatal myocardial infarction	4 (4.1)	7 (3.6)	0.826
Cardiovascular death	2 (2.0)	4 (2.1)	0.682
Stroke	11 (11.3)	13 (6.7)	0.175

Values are presented as numbers and precentages (%) and analyses were carried out using χ^2^ statistics. FMD: flow-mediated dilatation

**Figure 1:**
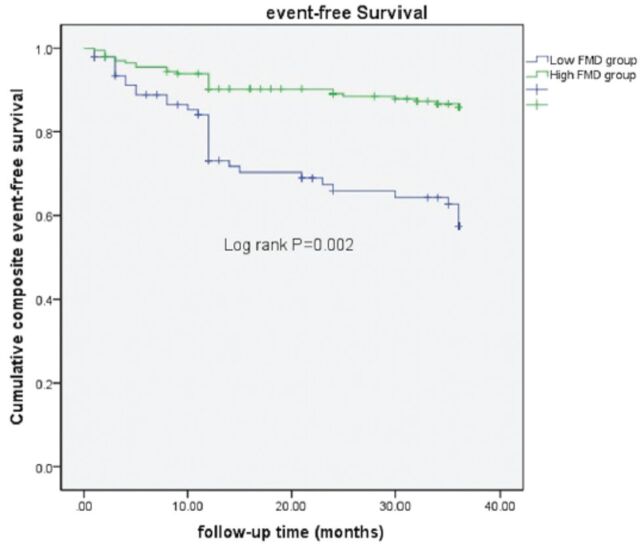
- Kaplan-Meier analysis for composite event free survival in both groups. FMD: flow-mediated dilatation

In a multivariate analysis using a Cox regression model, we demonstrated that low FMD (HR: 3.036, 95% CI: [1.546-5.963]), CHA2DS2-VASc scores (HR: 1.383, 95% CI: [1.035-1.847]), and LAD (HR: 1.304, 95% CI: [1.001-1.069]) were independent predictors for composite cardiovascular events (Table 3). As shown in Figure 1, a Kaplan-Meier survival curve demonstrated that patients with low FMD had significantly higher composite cardiovascular adverse endpoints compared with those with high FMD (*p*=0.002).

**Table 3:** - Cox proportional hazards model regression of composite cardiovascular events in patients with paroxysmal atrial fibrillation.

Characteristics	Univariate analysis		Multiple analysis	
	hazard ratio (95% CI)	*P*-value	hazard ratio (95% CI)	*P*-value
CHA2DS2-VASc score	1.718 (1.357-2.175)	0.000	1.383 (1.035-1.847)	0.028
Low FMD	3.371 (2.012-5.047)	0.000	3.036 (1.546-5.963)	0.001
LAD	1.048 (1.022-1.073)	0.000	1.034 (1.001-1.069)	0.041
LVD	1.027 (0.997-1.057)	0.076	0.979 (0.933-1.027)	0.389
LVEF	0.983 (0.965-1.000)	0.050	0.995 (0.967-1.023)	0.706
Smoking	1.138 (0.350-3.703)	0.739	---	---
CHD	0.841 (0.487-1.453)	0.535	---	---
TC	0.879 (0.680-1.136)	0.323	---	---
TG	0.844 (0.636-1.120)	0.240	---	---
LDL-C	0.948 (0.689-1.305)	0.744	---	---
Creatinine	1.044 (1.000-1.048)	0.038	1.001 (0.994-1.007)	0.798
Homocysteine	1.045 (1.018-1.072)	0.001	1.029 (0.999-1.060)	0.062
Wafarin user	0.883 (0.678-1.132)	0.036	0.913 (0.827-1.032)	0.412
NOAC user	0.956 (0.577-1.038)	0.116	---	---
Diuretics user	1.031 (0.780-1.237)	0.521	---	---
Beita blocker user	0.912 (0.650-1.332)	0.135	---	---
ACEI/ARB user	1.002 (0.676-1.567)	0.336	---	---

CI: confidence interval, CHA2DS2-VASc: congestive heart failure, hypertension, age ≥75 (doubled), diabetes, stroke (doubled), vascular disease, age 65-74, and gender category (female), FMD: flow-mediated dilatation, LAD: left atrial diameter, LVD: left ventricular diameter, LVEF: left ventricular ejection fraction, CHD: coronary artery disease, TC: totol cholesterol, TG: triglyceride, LDL-C: low density lipoprotein-cholesterol, NOAC: non-vitamin K antagonist oral anticoagulant, ACEI: angiotension converting enzyme inhibitors, ARB: angiotensin receptor blocker

**Figure 2:**
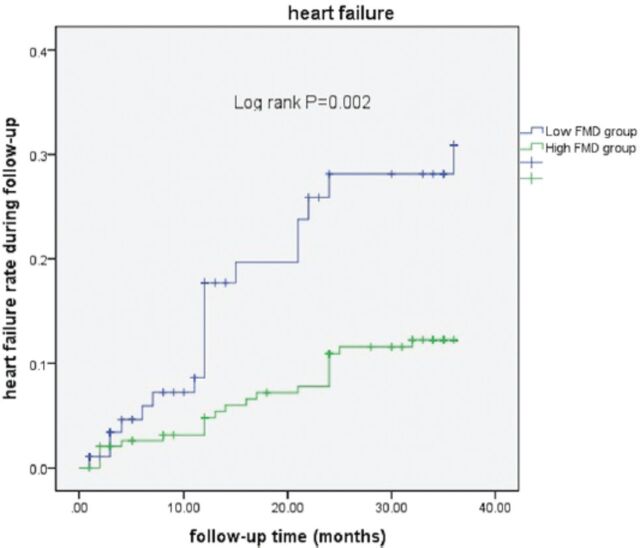
- Kaplan-Meier analysis for heart failure rate in both groups. FMD: flow-mediated dilatation

**Figure 3:**
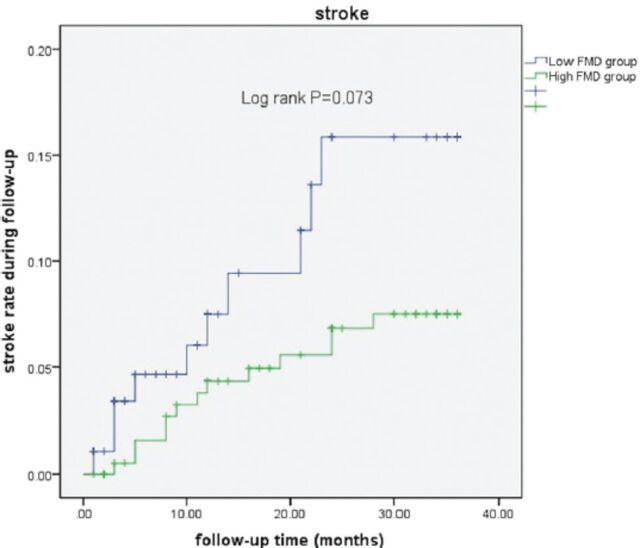
- Kaplan-Meier analysis for stroke in both groups. FMD: flow-mediated dilatation

Kaplan-Meier survival analysis also demonstrated that patients with low FMD had significantly higher heart failure endpoints compared with those with high FMD (*p*=0.002; Figure 2). The result of Cox regression showed that low FMD (HR: 3.2207, 95% CI: [1.320-7.7975]), homocysteine (HR: 1.048, 95% CI: [1.012-1.086]), and LAD (HR: 1.057, 95% CI: [1.022-1.093]) were independent predictors for composite cardiovascular events (Table 4). There was no significant difference of stroke endpoint between the 2 groups by Kaplan-Meier survival analysis (Figure 3).

**Table 4:** - Cox proportional hazards model regression of heart failure in patients with paroxysmal atrial fibrillation.

Characteristics	Univariate analysis		Multiple analysis	
	hazard ratio (95% CI)	*P*-value	hazard ratio (95% CI)	*P*-value
CHA2DS2-VASc score	1.214 (0.824-1.517)	0.086	1.204 (0.815-1.778)	0.352
Low FMD	2.651 (1.411-4.980)	0.002	3.207 (1.320-7.7975)	0.010
LAD	1.065 (1.037-1.093)	0.000	1.057 (1.022-1.093)	0.001
LVD	1.040 (1.009-1.072)	0.012	0.965 (0.915-1.019)	0.201
LVEF	0.978 (0.958-0.998)	0.028	0.992 (0.956-1.029)	0.654
Smoking	1.148 (0.650-2.703)	0.321	---	---
CHD	1.166 (0.570-2.386)	0.657	---	---
TC	0.814 (0.584-1.133)	0.212	---	---
TG	0.822 (0.563-1.198)	0.307	---	---
LDL-C	1.024 (0.698-1.880)	0.049	1.038 (0.638-1.688)	0.882
Creatinine	1.006 (1.0015-1.010)	0.009	1.002 (0.995-1.010)	0.588
Homocysteine	1.071 (1.042-1.102)	0.000	1.048 (1.012-1.086)	0.008
Wafarin user	0.893 (0.659-0.964)	0.045	0.897 (0.656-0.933)	0.121
NOAC user	0.935 (0.571-1.038)	0.139	---	---
Diuretics user	1.131 (0.770-1.245)	0.624	---	---
Beita blocker user	0.952 (0.671-1.358)	0.475	---	---
ACEI/ARB user	0.836 (0.456-0.932)	0.103	---	---

CI: confidence interval, CHA2DS2-VASc: congestive heart failure, hypertension, age ≥75 (doubled), diabetes, stroke (doubled), vascular disease, age 65-74, and gender category (female), FMD: flow-mediated dilatation, LAD: left atrial diameter, LVD: left ventricular diameter, LVEF: left ventricular ejection fraction, CHD: coronary artery disease, TC: totol cholesterol, TG: triglyceride, LDL-C: low density lipoprotein-cholesterol, NOAC: non-vitamin K antagonist oral anticoagulant, ACEI: angiotension converting enzyme inhibitors, ARB: angiotensin receptor blocker

## Discussion

This study demonstrated that low FMD was associated with cardiovascular events in patients with paroxysmal AF. Flow-mediated dilation was a significant predictor of cardiovascular events independent of conventional cardiovascular risks in patients with AF.

Healthy endothelium is a barrier between the bloodstream and the wall of the blood vessels and modulates vascular dilatation by paracrine cytokines such as nitric oxide and endothelin-1.^12^ Endothelial dysfunction plays a significant role in onset and development of cardiovascular diseases. Although risk factors such as diabetes, hypertension, smoke, and hyperlipemia are connected with the progression of cardiovascular disease, endothelial dysfunction maybe the final common pathway for these factors.^13^ In recent years, measurement of FMD by ultrasound has been used as a non-invasive method for assessment of endothelial function. Moreover, previous studies have shown that FMD could be used not just as a marker of endothelial function, but as a prognostic index of adverse events independent of conventional cardiovascular risk factors.^12-14^ However, most studies investigated the association between FMD and cardiovascular events in patients with coronary artery disease. In this prospective observational study, we found that a low FMD (FMD<5.9%) was an independent predictor of cardiovascular event in patients with AF. The cut-off value of FMD in this study was 5.9%. It was based on the highest value of Youden index from the receiver-operator characteristic curves for predicting the primary outcomes.

Cardiovascular events was primarily contributed by heart failure in this study. Heart failure is a major complication in AF patients. Mechanisms by which AF increases the risk of heart failure are not fully clarified. Impaired endothelial function maybe a possible mechanism by which AF complicates heart failure. It was well documented that patients with heart failure had impaired endothelial function.^13^ Previous studies also demonstrated that AF patients had decreased endothelial function.^4-6^ Our results indicated that patients with low FMD had a higher rate of heart failure hospitalization. There were several researches indicating that endothelial dysfunction caused decreased nitric oxide bioavailability and microvascular ischemia.^15,16^ Endothelial dysfunction could also increase inflammation and oxidative stress.^17^ All of these might contribute to heart failure in AF patients. As a result, endothelial dysfunction may play an important role in the development of heart failure in patients with paroxysmal AF.

### Study limitations

First of all, the cut-off value of low FMD in this study was 5.9%. It was based on the highest value of Youden index from the receiver-operator characteristic curves for predicting the primary outcomes. However, there is no unified standard to define impaired endothelial function or FMD. Further studies are needed to determine whether the cut-off values of FMD are universally valid. Second, we investigated paroxysmal AF patients and excluded persistent and permanent AF patients. These patients also have impaired endothelial function. We still need more studies to explore the relationship between impaired endothelial function and cardiovascular events in these patients.

In conclusion, baseline endothelial dysfunction was significantly associated with increased risk of cardiac events, especially heart failure. Consequently, endothelial dysfunction might have an important role in the development of cardiac dysfunction in AF patients.
